# Supplementary material for: Sex differences in the sympathetic neurocirculatory responses to chemoreflex activation
Source: J Physiol. 2022 May 12;600(11):2669–89. doi: 10.1113/JP282327 (PMC9324851; doi:10.1113/JP282327)
Supplement: Supplementary file 1 — Statistical Summary Document [file TJP-600-2669-s001.docx]

**Manuscript Title:** Sex-differences in the sympathetic neurocirculatory responses to chemoreflex activation.

**Authors:** Ana Luiza C Sayegh; Jui-Lin Fan; Lauro C Vianna; Mathew Dawes; John Kolbe; Julian F R Paton; James P Fisher.

**Animal model used, if applicable:**

**Underlying hypothesis:** Muscle sympathetic nerve activity responses to central chemoreflex activation (i.e., hypercapnic response) are enhanced in young women compared to young men.

**Definitions of ‘n’:** n = number of young healthy participants

Total young healthy participants: n=20; Women: n=10; Men: n=10.

For analyses of arterial baroreflex control of MSNA: total young healthy participants: n=18; Women: n=9; Men: n=9.

For analyses of spontaneous cardiac baroreflex sensitivity: total young healthy participants: n=15; Women: n=7; Men: n=8.

**Statistical summary table:**

| **Experimental question number*** | **Finding/ conclusion** | **Experimental location/ variable**  **e.g. cortex vs cerebellum or genotype** | **Mean value**  **(or other summary statistic)** | **SD** | **n (value)** | **Exact P value** | **Figure/table in which data are presented** | **Units** | **Data comparisons**  **e.g. WT vs KO** | **Statistical test** | **Any other variable**  **e.g. subjects’ age or sex** | **Comments**  **e.g. observation** |
| --- | --- | --- | --- | --- | --- | --- | --- | --- | --- | --- | --- | --- |
| Age was similar between women and men. | There were no sex-differences in age. | Age | **Age in:**  -Women: 29±5 years vs. Men: 30±7 years | Listed in previous column | Women: n=10; Men: n=10 | **P=0.615** | **-** | years | Women vs. Men | Students t-test |  |  |
| Subjects’ characteristic was different between women and men. | Weight and height were lower in women compared to men.  Body mass index (BMI) was similar between women and men. | Weight, height, and BMI in women and men | **Weight in:**  -Women: 64.2±9.1 kg vs. Men: 78.5±12.7 kg  **Height in:**  -Women: 1.68±0.8 m vs. Men: 1.78±0.6 m  **Body mass index in:**  -Women: 22.8±2.4 kg·m^2^ vs. Men: 24.8±3.2 kg·m^2^ | Same as above | Same as above | **Weight in women vs. men: P=0.010**  **Height in women vs. men: P=0.005**  **BMI in women vs. men: P=0.125** | **-** | Weight = kg  Height = m  BMI = kg·m^2^ | Same as above | Same as above |  |  |
| Partial pressure of end-tidal oxygen (P_ET_O_2_) was different between eucapnia, isocapnic hypoxia, hypercapnic hyperoxia, and hypercapnic hypoxia | Compared to eucapnia (room air), P_ET_O_2_ was decreased during isocapnic hypoxia and hypercapnic hypoxia and increased during hypercapnic hyperoxia | P_ET_O_2_ during eucapnia, isocapnic hypoxia, hypercapnic hyperoxia, and hypercapnic hypoxia | **P_ET_O_2_ during:**  - Eucapnia Women: 97±5 mmHg vs. Men: 96±9 mmHg  - Isocapnic hypoxia Women: 51±3 mmHg vs. Men: 50±5 mmHg  - Hypercapnic hyperoxia Women: 337±13 mmHg vs. Men: 348±20 mmHg  - Hypercapnic hypoxia Women: 60±3 mmHg vs. Men: 59±5 mmHg | Same as above | Same as above | **P_ET_O_2_ during:**  -Isocapnic hypoxia vs. eucapnia: **P<0.0001**  -Hypercapnic hyperoxia vs. eucapnia: **P<0.0001**  -Hypercapnic hypoxia vs. eucapnia: **P<0.0001**  -Isocapnic hypoxia vs. hypercapnic hyperoxia: **P<0.0001**  -Isocapnic hypoxia vs. hypercapnic hypoxia: **P=0.030**  **-**Hypercapnic hyperoxia vs. hypercapnic hypoxia: **P<0.0001** | Figure 2A | mmHg | Eucapnia vs. isocapnic hypoxia vs. hypercapnic hyperoxia vs. hypercapnic hypoxia | Mixed model analysis of variance (ANOVA) with repeated measures |  |  |
| Partial pressure of end-tidal oxygen (P_ET_O_2_) was similar between women and men during eucapnia, isocapnic hypoxia, hypercapnic hyperoxia, and hypercapnic hypoxia. | There were no sex-differences in the P_ET_O_2_ during eucapnia, isocapnic hypoxia, hypercapnic hyperoxia, and hypercapnic hypoxia. | P_ET_O_2_ in women and men during eucapnia, isocapnic hypoxia, hypercapnic hyperoxia, and hypercapnic hypoxia. | Same as above | Same as above | Same as above | **- P_ET_O_2_ in women vs. men:** **P=0.260** | Same as above | Same as above | Women vs. Men | Same as above |  |  |
| Partial pressure of end-tidal carbon dioxide (P_ET_CO_2_) was different between eucapnia, isocapnic hypoxia, hypercapnic hyperoxia, and hypercapnic hypoxia. | P_ET_CO_2_ was not different from eucapnia during isocapnic hypoxia but was increased during hypercapnic hyperoxia and hypercapnic hypoxia. | P_ET_CO_2_ during eucapnia, isocapnic hypoxia, hypercapnic hyperoxia, and hypercapnic hypoxia. | **P_ET_CO_2_ during:**  - Eucapnia Women: 41±2 mmHg vs. Men: 41±1 mmHg  - Isocapnic hypoxia Women: 40±2 mmHg vs. Men: 41±2 mmHg  - Hypercapnic hyperoxia Women: 55±3 mmHg vs. Men: 56±4 mmHg  - Hypercapnic hypoxia Women: 55±3 mmHg vs. Men: 56±3 mmHg | Same as above | Same as above | **P_ET_CO_2_ during:**  -Isocapnic hypoxia vs. eucapnia: **P=1.000**  -Hypercapnic hyperoxia vs. eucapnia: **P<0.0001**  -Hypercapnic hypoxia vs. eucapnia: **P<0.0001**  -Isocapnic hypoxia vs. hypercapnic hyperoxia: **P<0.0001**  -Isocapnic hypoxia vs. hypercapnic hypoxia: **P=0.030**  **-**Hypercapnic hyperoxia vs. hypercapnic hypoxia: **P=1.000** | Figure 2B | mmHg | Eucapnia vs. isocapnic hypoxia vs. hypercapnic hyperoxia vs. hypercapnic hypoxia | Same as above |  |  |
| Partial pressure of end-tidal carbon dioxide (P_ET_CO_2_) was similar between women and men during eucapnia, isocapnic hypoxia, hypercapnic hyperoxia, and hypercapnic hypoxia | There were no sex-differences in the P_ET_CO_2_ during eucapnia, isocapnic hypoxia, hypercapnic hyperoxia, and hypercapnic hypoxia | P_ET_CO_2_ in women and men during eucapnia, isocapnic hypoxia, hypercapnic hyperoxia, and hypercapnic hypoxia | Same as above | Same as above | Same as above | **P_ET_CO_2_ in women vs. men: P=0.115** | Same as above | Same as above | Women vs Men | Same as above |  |  |
| Oxygen saturation (S_P_O_2_) was different between eucapnia, isocapnic hypoxia, hypercapnic hyperoxia, and hypercapnic hypoxia. | Compared to eucapnia (room air), S_P_O_2_ was decreased during isocapnic hypoxia and hypercapnic hypoxia and increased during hypercapnic hyperoxia. | S_P_O_2_ during eucapnia, isocapnic hypoxia, hypercapnic hyperoxia, and hypercapnic hypoxia | **S_P_O_2_ during:**  - Eucapnia Women: 97±1 % vs. Men: 97±1 %  - Isocapnic hypoxia Women: 86±2 % vs. Men: 87±3 %  - Hypercapnic hyperoxia Women: 99±1 % vs. Men: 99±0 %  - Hypercapnic hypoxia Women: 89±1 % vs. Men: 92±2 % | Same as above | Same as above | **S_P_O_2_ during:**  -Isocapnic hypoxia vs. eucapnia: **P<0.0001**  -Hypercapnic hyperoxia vs. eucapnia: **P<0.0001**  -Hypercapnic hypoxia vs. eucapnia: **P<0.0001**  -Isocapnic hypoxia vs. hypercapnic hyperoxia: **P<0.0001**  -Isocapnic hypoxia vs. hypercapnic hypoxia: **P=0.030**  **-**Hypercapnic hyperoxia vs. hypercapnic hypoxia: **P<0.0001** | Table 1 | % | Eucapnia vs. isocapnic hypoxia vs. hypercapnic hyperoxia vs. hypercapnic hypoxia | Same as above |  |  |
| Oxygen saturation (S_P_O_2_) was similar between women and men during eucapnia, isocapnic hypoxia, hypercapnic hyperoxia, and hypercapnic hypoxia. | There were no sex-differences in S_P_O_2_ during eucapnia, isocapnic hypoxia, hypercapnic hyperoxia, and hypercapnic hypoxia. | S_P_O_2_ in women and men during eucapnia, isocapnic hypoxia, hypercapnic hyperoxia, and hypercapnic hypoxia. | Same as above | Same as above | Same as above | **S_P_O_2_ in women vs. men:** **P=0.109** | Same as above | Same as above | Women vs Men | Same as above |  |  |
| Respiratory variables (ventilation, V_E_; tidal volume, V_T_; and respiratory frequency, R*f*) were different between eucapnia, isocapnic hypoxia, hypercapnic hyperoxia, and hypercapnic hypoxia. | Hypercapnic hyperoxia and hypercapnic hypoxia increased V̇_E_, V̇_T_ and R*f* compared to eucapnia. | V̇_E_, V̇_T_, and R*f* during eucapnia, isocapnic hypoxia, hypercapnic hyperoxia, and hypercapnic hypoxia. | **V̇_E_ during:**  - Eucapnia Women: 12.1±1.9 L·min^-1^ vs. Men: 13.9±4.0 L·min^-1^  - Isocapnic hypoxia Women: 15.0±1.9 L·min^-1^ vs. Men: 17.3±5.0 L·min^-1^  - Hypercapnic hyperoxia Women: 27.3±6.3 L·min^-1^ vs. Men: 39.5±7.5L·min^-1^  - Hypercapnic hypoxia Women: 40.9±9.1 L·min^-1^ vs. Men: 53.8±13.3 L·min^-1^  **V̇_T_ during:**  - Eucapnia Women: 0.93±0.27 L vs. Men: 0.85±0.22 L  - Isocapnic hypoxia Women: 0.92±0.31 L vs. Men: 1.08±0.30 L  - Hypercapnic hyperoxia Women: 1.43±0.41 L vs. Men: 1.81±0.35 L  - Hypercapnic hypoxia Women: 1.73±0.41 L vs. Men: 2.06±0.39 L  **R*f* during:**  - Eucapnia Women: 13±5 breaths·min^-1^ vs. Men: 15±4 breaths·min^-1^  - Isocapnic hypoxia Women: 16±5 breaths·min^-1^ vs. Men: 16±5 breaths·min^-1^  - Hypercapnic hyperoxia Women: 18±5 breaths·min^-1^ vs. Men: 20±4 breaths·min^-1^  - Hypercapnic hypoxia Women: 22±5 breaths·min^-1^ vs. Men: 24±7 breaths·min^-1^ | Same as above | Same as above | **V̇_E_ comparison within women:**  -Isocapnic hypoxia vs. eucapnia: **P=1.000**  -Hypercapnic hyperoxia vs. eucapnia: **P<0.0001**  -Hypercapnic hypoxia vs. eucapnia: **P<0.0001**  -Isocapnic hypoxia vs. hypercapnic hyperoxia: **P=0.0001**  -Isocapnic hypoxia vs. hypercapnic hypoxia: **P<0.0001**  -Hypercapnic hyperoxia vs. hypercapnic hypoxia: **P=0.0001**  **V̇E comparison within men:**  -Isocapnic hypoxia vs. eucapnia: **P=1.000**  **-**Hypercapnic hyperoxia vs. eucapnia: **P<0.0001**  -Hypercapnic hypoxia vs. eucapnia: **P<0.0001**  -Isocapnic hypoxia vs. hypercapnic hyperoxia: **P<0.0001**  -Isocapnic hypoxia vs. hypercapnic hypoxia: **P<0.0001**  -Hypercapnic hyperoxia vs. hypercapnic hypoxia: **P=0.0004.**  **- V̇_T_** **during:**  -Isocapnic hypoxia vs. eucapnia: **P=0.110**  -Hypercapnic hyperoxia vs. eucapnia: **P<0.0001**  -Hypercapnic hypoxia vs. eucapnia: **P<0.0001**  -Isocapnic hypoxia vs. hypercapnic hyperoxia: **P<0.0001**  -Isocapnic hypoxia vs. hypercapnic hypoxia: **P<0.0001**  **-**Hypercapnic hyperoxia vs. hypercapnic hypoxia: **P=0.048**  **- R*f* during:**  -Isocapnic hypoxia vs. eucapnia: **P=0.397**  -Hypercapnic hyperoxia vs. eucapnia: **P<0.0001**  -Hypercapnic hypoxia vs. eucapnia: **P<0.0001**  -Isocapnic hypoxia vs. hypercapnic hyperoxia: **P=** **0.0120**  -Isocapnic hypoxia vs. hypercapnic hypoxia: **P<0.0001**  **-**Hypercapnic hyperoxia vs. hypercapnic hypoxia: **P=0.003** | Table 1, Figure 3 A and B | V̇_E_ = L·min^-1^  V̇_T_ = L  R*f* = breaths·min^-1^ | Eucapnia vs. isocapnic hypoxia vs. hypercapnic hyperoxia vs. hypercapnic hypoxia | Same as above |  |  |
| V̇_E_, V̇_T_, and R*f* were different between women and men during eucapnia, isocapnic hypoxia, hypercapnic hyperoxia, and hypercapnic hypoxia. | V̇_E_ and V̇_T_ were not different in women and men during eucapnia and isocapnic hypoxia. However, men showed augmented V̇_E_ and V̇_T_ during Hypercapnic hyperoxia and hypercapnic hypoxia compared to women.  There was no sex-difference in the R*f* during eucapnia, isocapnic hypoxia, hypercapnic hyperoxia, and hypercapnic hypoxia. | V̇_E_, V̇_T_, and R*f* in women and men during eucapnia, isocapnic hypoxia, hypercapnic hyperoxia, and hypercapnic hypoxia. | Same as above | Same as above | Same as above | **V̇_E_ in women vs. men during:**  **-** Eucapnia **P=0.587**  - Isocapnic hypoxia **P=0.471**  - Hypercapnic hyperoxia **P<0.0001**  - Hypercapnic hypoxia **P<0.0001**  **V̇_T_ in women vs. men during:**  **-** Eucapnia **P=0.637**  - Isocapnic hypoxia **P=0.278**  - Hypercapnic hyperoxia **P=0.018**  - Hypercapnic hypoxia **P=0.035**  **- R*f* in women vs. men: P=0.109** | Same as above | Same as above | Women vs Men | Same as above |  |  |
| The physiological sum of the V̇E responses to isocapnic hypoxia and hypercapnic hyperoxia was different than the responses to hypercapnic hypoxia. | The physiological sum of the V̇E responses to isocapnic hypoxia and hypercapnic hyperoxia was lower than the responses to hypercapnic hypoxia. | Physiological sum of the V̇E responses to isocapnic hypoxia and hypercapnic hyperoxia and the hypercapnic hypoxia response. | **Physiological sum of the V̇E responses to isocapnic hypoxia and hypercapnic hyperoxia:**  Women: 18.0±8.4 L·min^-1^ vs. Men: 29.0±9.5 L·min^-1^  **Hypercapnic hypoxia** Women: 28.8±9.3 L·min^-1^ vs. Men: 39.9±12.4 L·min^-1^ | Same as above | Same as above | **Physiological sum of the V̇E responses to isocapnic hypoxia and hypercapnic hyperoxia vs. hypercapnic hypoxia:**  **P=<0.0001** | Figure 3B | L·min^-1^ | Physiological sum of the V̇E responses to isocapnic hypoxia and hypercapnic hyperoxia | Same as above |  |  |
| The physiological sum of the V̇E responses to isocapnic hypoxia and hypercapnic hyperoxia was different than the responses to hypercapnic hypoxia in women and men. | Men showed an augmented response in V̇E during hypercapnic hypoxia than the response to the physiological sum of the V̇E to isocapnic hypoxia and hypercapnic hyperoxia compared to women. | Physiological sum of the V̇E responses to isocapnic hypoxia and hypercapnic hyperoxia and the hypercapnic hypoxia response in women and men. | Same as above | Same as above | Same as above | **Physiological sum of the V̇E responses to isocapnic hypoxia and hypercapnic hyperoxia in women and men:**  **P=0.015** | Same as above | Same as above | Physiological sum of the V̇E responses to isocapnic hypoxia and hypercapnic hyperoxia | Same as above |  |  |
| Perception of breathlessness was different between eucapnia, isocapnic hypoxia, hypercapnic hyperoxia, and hypercapnic hypoxia. | The perception of breathlessness increased progressively from the isocapnic hypoxia, hypercapnic hyperoxia, and hypercapnic hypoxia trials. | Perception of breathlessness during eucapnia, isocapnic hypoxia, hypercapnic hyperoxia, and hypercapnic hypoxia. | **Perception of breathlessness during:**  - Eucapnia Women: 0±0 a.u. vs. Men: 0±0 a.u.  - Isocapnic hypoxia Women: 1±1 a.u. vs. Men: 1±1 a.u.  - Hypercapnic hyperoxia Women: 5±2 a.u. vs. Men: 5±1 a.u.  - Hypercapnic hypoxia Women: 7±2 a.u. vs. Men: 7±2 a.u. | Same as above | Same as above | **Perception of breathlessness:**  -Isocapnic hypoxia vs. eucapnia: **P=1.000**  -Hypercapnic hyperoxia vs. eucapnia: **P<0.0001**  -Hypercapnic hypoxia vs. eucapnia: **P<0.0001**  -Isocapnic hypoxia vs. hypercapnic hyperoxia: **P<0.0001**  -Isocapnic hypoxia vs. hypercapnic hypoxia: **P=0.030**  **-**Hypercapnic hyperoxia vs. hypercapnic hypoxia: **P<0.0001** | Table 1 | a.u. | Eucapnia vs. isocapnic hypoxia vs. hypercapnic hyperoxia vs. hypercapnic hypoxia | Same as above |  |  |
| Perception of breathlessness was similar between women and men during eucapnia, isocapnic hypoxia, hypercapnic hyperoxia, and hypercapnic hypoxia. | There were no sex-differences in the perception of breathlessness during eucapnia, isocapnic hypoxia, hypercapnic hyperoxia, and hypercapnic hypoxia. | Perception of breathlessness in women and men during eucapnia, isocapnic hypoxia, hypercapnic hyperoxia, and hypercapnic hypoxia. | Same as above | Same as above | Same as above | **Perception of breathlessness in women vs. men:** **P=0.699** | Same as above | Same as above | Women vs. Men | Same as above |  |  |
| Cardiovascular variables (heart rate, HR; systolic blood pressure, SBP; diastolic blood pressure, DBP; mean arterial pressure, MAP; cardiac output, CO; cardiac output index, COi; stroke volume, SV; total peripheral resistance, TPR; and total peripheral resistance index, TPRi) were different between eucapnia, isocapnic hypoxia, hypercapnic hyperoxia, and hypercapnic hypoxia. | Compared to eucapnia, HR was similarly elevated during isocapnic hypoxia and hypercapnic hypoxia.  MAP was increased during isocapnic hypoxia, hypercapnic hyperoxia, and hypercapnic hypoxia compared to eucapnia.  CO and COi increased during isocapnic hypoxic and hypercapnic hypoxia compared to eucapnia.  TPR and TPRi increased only during hypercapnic hypoxia compared to eucapnia. | HR, SBP, DBP, MAP, CO, COi, SV, TPR, and TPRi during eucapnia, isocapnic hypoxia, hypercapnic hyperoxia and hypercapnic hypoxia | **HR during:**  - Eucapnia Women: 70±10 beats·min-^1^ vs. Men: 69±7 beats·min-^1^  - Isocapnic hypoxia Women: 78±10 beats·min-^1^ vs. Men: 75±5 beats·min-^1^  - Hypercapnic hyperoxia Women: 73±9 beats·min-^1^ vs. Men: 73±6 beats·min-^1^  - Hypercapnic hypoxia Women: 84±12 beats·min-^1^ vs. Men: 83±8 beats·min-^1^  **SBP during:**  - Eucapnia Women: 113±10 mmHg vs. Men: 126±10 mmHg  - Isocapnic hypoxia Women: 122±9 mmHg vs. Men: 134±8 mmHg  - Hypercapnic hyperoxia Women: 125±15 mmHg vs. Men: 141±14 mmHg  - Hypercapnic hypoxia Women: 143±17 mmHg vs. Men: 153±18 mmHg  **DBP during:**  - Eucapnia Women: 71±7 mmHg vs. Men: 68±12 mmHg  - Isocapnic hypoxia Women: 75±5 mmHg vs. Men: 75±13 mmHg  - Hypercapnic hyperoxia Women: 79±10 mmHg vs. Men: 82±14 mmHg  - Hypercapnic hypoxia Women: 89±9 mmHg vs. Men: 88±12 mmHg  **MAP during:**  - Eucapnia Women: 85±7 mmHg vs. Men: 88±12 mmHg  - Isocapnic hypoxia Women: 92±5 mmHg vs. Men: 98±11 mmHg  - Hypercapnic hyperoxia Women: 96±11 mmHg vs. Men: 104±15 mmHg  - Hypercapnic hypoxia Women: 108±11 mmHg vs. Men: 112±12 mmHg  **CO during:**  - Eucapnia Women: 5.6±1.7 L·min^-1^ vs. Men: 5.9±1.2 L·min^-1^  - Isocapnic hypoxia Women: 6.2±1.6 L·min^-1^ vs. Men: 6.8±1.5 L·min^-1^  - Hypercapnic hyperoxia Women: 5.8±1.6 L·min^-1^ vs. Men: 6.6±1.7 L·min^-1^  - Hypercapnic hypoxia Women: 7.0±2.5 L·min^-1^ vs. Men: 7.2±1.8 L·min^-1^  **COi during:**  - Eucapnia Women: 3.3±1.1 L·min^-1^·m^-2^ vs. Men: 3.1±0.7 L·min^-1^·m^-2^  - Isocapnic hypoxia Women: 3.6±1.0 L·min^-1^·m^-2^ vs. Men: 3.5±0.9 L·min^-1^·m^-2^  - Hypercapnic hyperoxia Women: 3.4±1.1 L·min^-1^·m^-2^ vs. Men: 3.4±1.0 L·min^-1^·m^-2^  - Hypercapnic hypoxia Women: 4.1±1.5 L·min^-1^·m^-2^ vs. Men: 3.7±1.1 L·min^-1^·m^-2^  **SV during:**  - Eucapnia Women: 84±20 mL vs. Men: 87±18 mL  - Isocapnic hypoxia Women: 82±22 mL vs. Men: 90±18 mL  - Hypercapnic hyperoxia Women: 83±21 mL vs. Men: 91±23 mL  - Hypercapnic hypoxia Women: 86±28 mL vs. Men: 86±20 mL  **TPR during:**  - Eucapnia Women: 16.5±5.4 mmHg·L^-1^·min^-1^ vs. Men: 15.6±4.5 mmHg·L^-1^·min^-1^  - Isocapnic hypoxia Women: 16.2±5.6 mmHg·L^-1^·min^-1^ vs. Men: 15.4±5.4 mmHg·L^-1^·min^-1^  - Hypercapnic hyperoxia Women: 18.3±8.0 mmHg·L^-1^·min^-1^ vs. Men: 17.3±7.1 mmHg·L^-1^·min^-1^  - Hypercapnic hypoxia Women: 17.6±8.0 mmHg·L^-1^·min^-1^ vs. Men: 16.7±5.0 mmHg·L^-1^·min^-1^  **TPRi during:**  - Eucapnia Women: 9.5±2.7 mmHg·L^-1^·min^-1^·m^-2^ vs. Men: 7.9±2.1 mmHg·L^-1^·min^-1^·m^-2^  - Isocapnic hypoxia Women: 9.3±2.7 mmHg·L^-1^·min^-1^·m^-2^ vs. Men: 7.8±2.5 mmHg·L^-1^·min^-1^·m^-2^  - Hypercapnic hyperoxia Women: 10.5±3.8 mmHg·L^-1^·min^-1^·m^-2^ vs. Men: 8.8±3.3 mmHg·L^-1^·min^-1^·m^-2^  - Hypercapnic hypoxia Women: 10.1±3.9 mmHg·L^-1^·min^-1^·m^-2^ vs. Men: 8.5±2.2 mmHg·L^-1^·min^-1^·m^-2^ | Same as above | Same as above | **- HR during:**  -Isocapnic hypoxia vs. eucapnia: **P<0.0001**  -Hypercapnic hyperoxia vs. eucapnia: **P=0.068**  -Hypercapnic hypoxia vs. eucapnia: **P<0.0001**  -Isocapnic hypoxia vs. hypercapnic hyperoxia: **P=0.022**  -Isocapnic hypoxia vs. hypercapnic hypoxia: **P<0.0001**  **-**Hypercapnic hyperoxia vs. hypercapnic hypoxia: **P<0.0001**  **- SBP** **during:**  -IIsocapnic hypoxia vs. eucapnia: **P=0.033**  -Hypercapnic hyperoxia vs. eucapnia: **P<0.0001**  -Hypercapnic hypoxia vs. eucapnia: **P<0.0001**  -Isocapnic hypoxia vs. hypercapnic hyperoxia: **P=0.740**  -Isocapnic hypoxia vs. hypercapnic hypoxia: **P<0.0001**  **-**Hypercapnic hyperoxia vs. hypercapnic hypoxia: **P<0.0001**  **- DBP during:**  -Isocapnic hypoxia vs. eucapnia: **P=0.074**  -Hypercapnic hyperoxia vs. eucapnia: **P<0.0001**  -Hypercapnic hypoxia vs. eucapnia: **P<0.0001**  -Isocapnic hypoxia vs. hypercapnic hyperoxia: **P=0.038**  -Isocapnic hypoxia vs. hypercapnic hypoxia: **P<0.0001**  **-**Hypercapnic hyperoxia vs. hypercapnic hypoxia: **P=0.001**  **- MAP during:**  -Isocapnic hypoxia vs. eucapnia: **P<0.0001**  -Hypercapnic hyperoxia vs. eucapnia: **P<0.0001**  -Hypercapnic hypoxia vs. eucapnia: **P<0.0001**  -Isocapnic hypoxia vs. hypercapnic hyperoxia: **P=0.095**  -Isocapnic hypoxia vs. hypercapnic hypoxia: **P<0.0001**  **-**Hypercapnic hyperoxia vs. hypercapnic hypoxia: **P<0.0001**  **- CO during:**  -Isocapnic hypoxia vs. eucapnia: **P=0.002**  -Hypercapnic hyperoxia vs. eucapnia: **P=0.145**  -Hypercapnic hypoxia vs. eucapnia: **P<0.0001**  -Isocapnic hypoxia vs. hypercapnic hyperoxia: **P=0.719**  -Isocapnic hypoxia vs. hypercapnic hypoxia: **P=0.006**  **-**Hypercapnic hyperoxia vs. hypercapnic hypoxia: **P<0.0001**  **- COi during:**  -Isocapnic hypoxia vs. eucapnia: **P=0.002**  -Hypercapnic hyperoxia vs. eucapnia: **P=0.146**  -Hypercapnic hypoxia vs. eucapnia: **P<0.0001**  -Isocapnic hypoxia vs. hypercapnic hyperoxia: **P=0.862**  -Isocapnic hypoxia vs. hypercapnic hypoxia: **P=0.003**  **-**Hypercapnic hyperoxia vs. hypercapnic hypoxia: **P<0.0001**  **- SV**  **P=0.939**  **- TPR during:**  -Isocapnic hypoxia vs. eucapnia: **P=1.000**  -hypercapnic hyperoxia vs. eucapnia: **P=0.028**  -Hypercapnic hypoxia vs. eucapnia: **P=0.400**  -Isocapnic hypoxia vs. hypercapnic hyperoxia: **P=0.008**  -Isocapnic hypoxia vs. hypercapnic hypoxia: **P=0.150**  **-**Hypercapnic hyperoxia vs. hypercapnic hypoxia: **P=1.000**  **- TPRi during:**  -Isocapnic hypoxia vs. eucapnia: **P=1.000**  -Hypercapnic hyperoxia vs. eucapnia: **P=0.030**  -Hypercapnic hypoxia vs. eucapnia: **P=0.424**  -Isocapnic hypoxia vs. hypercapnic hyperoxia: **P=0.008**  -Isocapnic hypoxia vs. hypercapnic hypoxia: **P=0.155**  **-**Hypercapnic hyperoxia vs. hypercapnic hypoxia: **P=1.000** | Table 1 | HR = beats·min^-1^  SBP = mmHg  DBP = mmHg  MAP = mmHg  CO = L·min^-1^  COi = L·min^-1^·m^-2^  SV = mL  TPR = mmHg·L^-1^·min^-1^  TPRi = mmHg·L^-1^·min^-1^·m^-2^ | Eucapnia vs. isocapnic hypoxia vs. hypercapnic hyperoxia vs. hypercapnic hypoxia | Same as above |  |  |
| Cardiovascular variables (heart rate, HR; systolic blood pressure, SBP; diastolic blood pressure, DBP; mean arterial pressure, MAP; cardiac output, CO; cardiac output index, COi; stroke volume, SV; total peripheral resistance, TPR; and total peripheral resistance index, TPRi) were different between women and men during eucapnia, isocapnic hypoxia, hypercapnic hyperoxia, and hypercapnic hypoxia. | There were no sex-differences in HR, DBP, MAP, CO, COi, SV, TPR, and TPRi during eucapnia, isocapnic hypoxia, hypercapnic hyperoxia, and hypercapnic hypoxia  SBP was lower in women during eucapnia, isocapnic hypoxia, hypercapnic hyperoxia, and hypercapnic hypoxia compared to men. | HR, SBP, DBP, MAP, CO, COi, SV, TPR, and TPRi in women and men during eucapnia, isocapnic hypoxia, hypercapnic hyperoxia, and hypercapnic hypoxia. | Same as above | Same as above | Same as above | **HR in women vs. men:** **P=0.706**  **- DBP in women vs. men:** **P=0.861**  **- MAP in women vs. men:** **P=0.265**  **- CO in women vs. men:** **P=0.543**  **- COi in women vs. men:** **P=0.667**  **- SV in women vs. men:** **P=0.582**  **- TPR in women vs. men:** **P=0.742**  **- TPRi in women vs. men:** **P=0.292**  **SBP in women vs. men during:**  **-** Eucapnia **P=** **0.011**  - Isocapnic hypoxia **P=** **0.010**  - Hypercapnic hyperoxia **P=0.021**  - Hypercapnic hypoxia **P=0.015** | Same as above | Same as above | Women vs. Men | Same as above |  |  |
| Muscle sympathetic nerve activity (MSNA) in burst frequency (BF), burst incidence (BI), burst amplitude, and total MSNA were different between eucapnia, isocapnic hypoxia, hypercapnic hyperoxia, and hypercapnic hypoxia. | MSNA in BF, BI, burst amplitude, and total MSNA were increased during hypercapnic hyperoxia and hypercapnic hypoxia compared to eucapnia. | MSNA during eucapnia, isocapnic hypoxia, hypercapnic hyperoxia, and hypercapnic hypoxia. | **MSNA in BF during:**  - Eucapnia Women: 11±3 bursts·min^-1^ vs. Men: 11±5 bursts ·min^-1^  - Isocapnic hypoxia Women: 15±4 bursts·min^-1^ vs. Men: 15±5 bursts ·min^-1^  - Hypercapnic hyperoxia Women: Women: 22±6 bursts·min^-1^ vs. Men: 19±6 bursts ·min^-1^  - Hypercapnic hypoxia Women: 30±8 bursts·min^-1^ vs. Men: 24±6 bursts ·min^-1^  **MSNA in BI during:**  - Eucapnia Women: 16±2 bursts·100 heartbeats^-1^ vs. Men: 17±2 bursts ·100 heartbeats^-1^  - Isocapnic hypoxia Women: 19±2 bursts·100 heartbeats^-1^ vs. Men: 20±2 bursts ·100 heartbeats^-1^  - Hypercapnic hyperoxia Women: 30±3 bursts·100 heartbeats^-1^ vs. Men: 25±2 bursts ·100 heartbeats^-1^  - Hypercapnic hypoxia Women: 36±3 bursts·100 heartbeats^-1^ vs. Men: 30±2 bursts ·100 heartbeats^-1^  **MSNA in burst amplitude during:**  - Eucapnia Women: 100±0 % vs. Men: 100±0 %  - Isocapnic hypoxia Women: 119±35 % vs. Men: 131±45 %  - Hypercapnic hyperoxia Women: 195±34 % vs. Men: 147±56 %  - Hypercapnic hypoxia Women: 218±63 % vs. Men: 170±64 %  **Total MSNA during:**  - Eucapnia Women: 100±0 % vs. Men: 100±0 %  - Isocapnic hypoxia Women: 151±61 % vs. Men: 140±36 %  - Hypercapnic hyperoxia Women: 378±215 % vs. Men: 257±107 %  - Hypercapnic hypoxia Women: 607±290 % vs. Men: 362±268 % | Same as above | Same as above | **MSNA in BF:**  -Isocapnic hypoxia vs. eucapnia: **P=0.209**  -Hypercapnic hyperoxia vs. eucapnia: **P<0.0001**  -Hypercapnic hypoxia vs. eucapnia: **P<0.0001**  -Isocapnic hypoxia vs. hypercapnic hyperoxia: **P<0.0001**  -Isocapnic hypoxia vs. hypercapnic hypoxia: **P<0.0001**  **-**Hypercapnic hyperoxia vs. hypercapnic hypoxia: **P<0.0001**  **- MSNA in BI:**  -Isocapnic hypoxia vs. eucapnia: **P=0.502**  -Hypercapnic hyperoxia vs. eucapnia: **P<0.0001**  -Hypercapnic hypoxia vs. eucapnia: **P<0.0001**  -Isocapnic hypoxia vs. hypercapnic hyperoxia: **P=0.012**  -Isocapnic hypoxia vs. hypercapnic hypoxia: **P<0.0001**  **-**Hypercapnic hyperoxia vs. hypercapnic hypoxia: **P=0.049**  **- MSNA in burst amplitude:**  -Isocapnic hypoxia vs. eucapnia: **P=1.000**  -Hypercapnic hyperoxia vs. eucapnia: **P<0.0001**  -Hypercapnic hypoxia vs. eucapnia: **P<0.0001**  -Isocapnic hypoxia vs. hypercapnic hyperoxia: **P=0.005**  -Isocapnic hypoxia vs. hypercapnic hypoxia: **P<0.0001**  **-**Hypercapnic hyperoxia vs. hypercapnic hypoxia: **P=1.000**  **- Total MSNA comparison within women:**  -Isocapnic hypoxia vs. eucapnia: **P=1.000**  -Hypercapnic hyperoxia vs. eucapnia: **P<0.0001**  -Hypercapnic hypoxia vs. eucapnia: **P<0.0001**  -Isocapnic hypoxia vs. hypercapnic hyperoxia: **P=0.002**  -Isocapnic hypoxia vs. hypercapnic hypoxia: **P<0.0001**  -Hypercapnic hyperoxia vs. hypercapnic hypoxia: **P=0.0003**  **total MSNA comparison within men:**  **-**Isocapnic hypoxia vs. eucapnia: **P=1.000**  -Hypercapnic hyperoxia vs. eucapnia: **P=0.01**  -Hypercapnic hypoxia vs. eucapnia: **P=0.003**  -Isocapnic hypoxia vs. hypercapnic hyperoxia: **P=0.02**  -Isocapnic hypoxia vs. hypercapnic hypoxia: **P=0.015**  -Hypercapnic hyperoxia vs. hypercapnic hypoxia: **P=0.03** | Table 1, Figure 3C and D | MSNA in BF = bursts·min^-1^  MSNA in BI = bursts·100 heartbeats^-1^  MSNA in burst amplitude = %  Total MSNA = % | Eucapnia vs. isocapnic hypoxia vs. hypercapnic hyperoxia vs. hypercapnic hypoxia | Same as above |  |  |
| Muscle sympathetic nerve activity (MSNA) in burst frequency (BF), burst incidence (BI), burst amplitude, and total MSNA were different between women and men during eucapnia, isocapnic hypoxia, hypercapnic hyperoxia, and hypercapnic hypoxia. | MSNA in BF and BI were not different in women and men during eucapnia.  Women showed an increased MSNA in BF during hypercapnic hypoxia, MSNA in burst amplitude during hypercapnic hyperoxia and hypercapnic hypoxia, and total MSNA during hypercapnic hyperoxia and hypercapnic hypoxia compared to men. | MSNA in women and men during eucapnia, isocapnic hypoxia, hypercapnic hyperoxia, and hypercapnic hypoxia. | Same as above | Same as above | Same as above | **MSNA in BF in women vs. men during:**  **-** Eucapnia **P=0.799**  - Isocapnic hypoxia **P=0.717**  - Hypercapnic hyperoxia **P=0.393**  - Hypercapnic hypoxia **P=0.034**  **MSNA in BI in women vs. men during:**  **-** Eucapnia **P=0.765**  - Isocapnic hypoxia **P=0.643**  - Hypercapnic hyperoxia **P=0.396**  - Hypercapnic hypoxia **P=0.041**  **MSNA in burst amplitude in women vs. men during:**  **-** Eucapnia **P=1.000**  - Isocapnic hypoxia **P=0.541**  - Hypercapnic hyperoxia **P=0.018**  - Hypercapnic hypoxia **P=0.017**  **Total MSNA in women vs. men during:**  **-** Eucapnia **P=1.000**  - Isocapnic hypoxia **P=0.616**  - Hypercapnic hyperoxia **P=0.017**  - Hypercapnic hypoxia **P<0.0001** | Same as above | Same as above | Women vs Men | Same as above |  |  |
| The physiological sum of the total MSNA responses to isocapnic hypoxia and hypercapnic hyperoxia was different than the responses to hypercapnic hypoxia. | The physiological sum of the total MSNA responses to isocapnic hypoxia and hypercapnic hyperoxia was lower than the responses to hypercapnic hypoxia. | Physiological sum of the total MSNA responses to isocapnic hypoxia and hypercapnic hyperoxia and the hypercapnic hypoxia response. | **Physiological sum of the total MSNA responses to isocapnic hypoxia and hypercapnic hyperoxia:**  Women: 328±224 % vs. Men: 197±118 %  **Hypercapnic hypoxia** Women: 507±290 % vs. Men: 262±268 % | Same as above | Same as above | **Physiological sum of the total MSNA responses to isocapnic hypoxia and hypercapnic hyperoxia vs. hypercapnic hypoxia:**  **P=0.048** | Figure 3D | % | Physiological sum of the total MSNA responses to isocapnic hypoxia and hypercapnic hyperoxia | Same as above |  |  |
| The physiological sum of the total MSNA responses to isocapnic hypoxia and hypercapnic hyperoxia was different than the responses to hypercapnic hypoxia in women and men. | Men showed an augmented response in total MSNA during hypercapnic hypoxia than the response to the physiological sum of the total MSNA to isocapnic hypoxia and hypercapnic hyperoxia compared to women. | Physiological sum of the total MSNA responses to isocapnic hypoxia and hypercapnic hyperoxia and the hypercapnic hypoxia response in women and men. | Same as above | Same as above | Same as above | **Physiological sum of the total MSNA responses to isocapnic hypoxia and hypercapnic hyperoxia in women and men:**  **P=0.044** | Same as above | Same as above | Physiological sum of the total MSNA responses to isocapnic hypoxia and hypercapnic hyperoxia | Same as above |  |  |
| Baroreflex sensitivity was different between eucapnia, isocapnic hypoxia, hypercapnic hyperoxia, and hypercapnic hypoxia. | Arterial baroreflex control of total MSNA (ABR-MSNA), but not burst incidence, was increased during hypercapnic hyperoxia compared to eucapnia. | ABR-MSNA during eucapnia, isocapnic hypoxia, hypercapnic hyperoxia, and hypercapnic hypoxia. | **ABR-MSNA in burst incidence during:**  - Eucapnia Women: -4.14±1.45 bursts [100 heartbeats]^-1^·mmHg^-1^ vs. Men: -3.80±1.54 bursts [100 heartbeats]^-1^·mmHg^-1^  - Isocapnic hypoxia Women: -3.25±1.20 bursts [100 heartbeats]^-1^·mmHg^-1^ vs. Men: -3.47±1.59 bursts [100 heartbeats]^-1^·mmHg^-1^  - Hypercapnic hyperoxia Women: -4.05±1.90 bursts [100 heartbeats]^-1^·mmHg^-1^ vs. Men: -4.51±1.84 bursts [100 heartbeats]^-1^·mmHg^-1^  - Hypercapnic hypoxia Women: -3.24±2.11 bursts [100 heartbeats]^-1^·mmHg^-1^ vs. Men: -3.35±2.01 bursts [100 heartbeats]^-1^·mmHg^-1^  **ABR-MSNA in Total MSNA during:**  - Eucapnia Women: -2.50±0.81 a.u.·beat^-1^·mmHg^-1^ vs. Men: -2.21±1.00 a.u.·beat^-1^·mmHg^-1^  - Isocapnic hypoxia Women: -2.45±1.01 a.u.·beat^-1^·mmHg^-1^ vs. Men: -2.67±1.15 a.u.·beat^-1^·mmHg^-1^  - Hypercapnic hyperoxia Women: -4.42±2.34 a.u.·beat^-1^·mmHg^-1^ vs. Men: -3.61±2.19 a.u.·beat^-1^·mmHg^-1^  - Hypercapnic hypoxia Women: -3.35±2.07 a.u.·beat^-1^·mmHg^-1^ vs. Men: -3.24±1.49 a.u.·beat^-1^·mmHg^-1^ | Same as above | Women: n=9; Men: n=9 | **ABR-MSNA:**  **- in BI:** **P=0.076**  **- in Total MSNA:**  - Isocapnic hypoxia vs. eucapnia: **P=1.000**  -Hypercapnic hyperoxia vs. eucapnia: **P=0.012**  -Hypercapnic hypoxia vs. eucapnia: **P=0.696**  -Isocapnic hypoxia vs. hypercapnic hyperoxia: **P=0.039**  -Isocapnic hypoxia vs. hypercapnic hypoxia: **P=1.000**  **-**Hypercapnic hyperoxia vs. hypercapnic hypoxia: **P=0.775** | Table 2, Figure 4 | ABR-MSNA in burst incidence = bursts [100 heartbeats]^-1^·mmHg^-1^  ABR-MSNA in Total MSNA = a.u.·beat^-1^·mmHg^-1^ | Eucapnia vs. isocapnic hypoxia vs. hypercapnic hyperoxia vs. hypercapnic hypoxia | Same as above |  |  |
| Baroreflex sensitivity was different between women and men during eucapnia, isocapnic hypoxia, hypercapnic hyperoxia, and hypercapnic hypoxia. | ABR-MSNA was not different between women and men. | ABR-MSNA in women and men during eucapnia, isocapnic hypoxia, hypercapnic hyperoxia, and hypercapnic hypoxia. | Same as above | Same as above | Same as above | **ABR-MSNA:**  **- in BI in women vs. men:**  **P=0.903**  **in total MSNA in women vs. men:**  **P=0.525** | Same as above | Same as above | Women vs Men | Same as above |  |  |
| Spontaneous cardiac baroreflex sensitivity (cBRS) was different between eucapnia, isocapnic hypoxia, hypercapnic hyperoxia, and hypercapnic hypoxia. | cBRS gain was increased during hypercapnic hyperoxia compared to hypercapnic hypoxia.  Baroreflex sequences and baroreflex effectiveness index (BEI) were decreased during hypercapnic hyperoxia and hypercapnic hypoxia compared to eucapnia. | cBRS during eucapnia, isocapnic hypoxia, hypercapnic hyperoxia, and hypercapnic hypoxia | **cBRS in gain during:**  - Eucapnia Women: 15.6±5.0 ms·mmHg^-1^ vs. Men: 14.2±5.2 ms·mmHg^-1^  - Isocapnic hypoxia Women: 10.7±4.5 ms·mmHg^-1^ vs. Men: 14.7±14.9 ms·mmHg^-1^  - Hypercapnic hyperoxia Women: 14.4±6.1 ms·mmHg^-1^ vs. Men: 19.5±10.1 ms·mmHg^-1^  - Hypercapnic hypoxia Women: 12.0±6.0 ms·mmHg^-1^ vs. Men: 14.1±10.2 ms·mmHg^-1^  cBRS in number of sequences during:  - Eucapnia Women: 33±20 n vs. Men: 23±17 n  - Isocapnic hypoxia Women: 33±18 n vs. Men: 21±18 a.u.·beat^-1^·mmHg^-1^  - Hypercapnic hyperoxia Women: 18±17 n vs. Men: 10±7 n  - Hypercapnic hypoxia Women: 25±18 n vs. Men: 8±11 n  cBRS in BEI during:  - Eucapnia Women: 0.57±0.32 % vs. Men: 0.50±0.25 %  - Isocapnic hypoxia Women: 0.53±0.26 % vs. Men: 0.38±0.33 %  - Hypercapnic hyperoxia Women: 0.35±0.25 % vs. Men: 0.35±0.28 %  - Hypercapnic hypoxia Women: 0.26±0.15 % vs. Men: 0.19±0.11 % | Same as above | Women: n=7; Men: n=8 | **cBRS**  **in gain:**  - Isocapnic hypoxia vs. eucapnia: **P=1.000**  -Hypercapnic hyperoxia vs. eucapnia: **P=1.000**  -Hypercapnic hypoxia vs. eucapnia: **P=0.319**  -Isocapnic hypoxia vs. hypercapnic hyperoxia: **P=0.577**  -Isocapnic hypoxia vs. hypercapnic hypoxia: **P=1.000**  **-**Hypercapnic hyperoxia vs. hypercapnic hypoxia: **P=0.045**  **in number of sequences:**  - Isocapnic hypoxia vs. eucapnia: **P=1.000**  -Hypercapnic hyperoxia vs. eucapnia: **P=0.014**  -hypercapnic hypoxia vs. eucapnia: **P=0.002**  -Isocapnic hypoxia vs. hypercapnic hyperoxia: **P=0.027**  -Isocapnic hypoxia vs. hypercapnic hypoxia: **P=0.003**  **-**Hypercapnic hyperoxia vs. hypercapnic hypoxia: **P=1.000**  **in BEI:**  - Isocapnic hypoxia vs. eucapnia: **P=1.000**  -Hypercapnic hyperoxia vs. eucapnia: **P=0.046**  -Hypercapnic hypoxia vs. eucapnia: **P<0.0001**  -Isocapnic hypoxia vs. hypercapnic hyperoxia: **P=0.677**  -Isocapnic hypoxia vs. hypercapnic hypoxia: **P=0.009**  **-**Hypercapnic hyperoxia vs. hypercapnic hypoxia: **P=0.471** | Table 2, Figure 4 | cBRS in gain = ms·mmHg^-1^  cBRS in number of sequences = n  cBRS in BEI = % | Eucapnia vs. isocapnic hypoxia vs. hypercapnic hyperoxia vs. hypercapnic hypoxia | Same as above |  |  |
| Spontaneous cardiac baroreflex sensitivity (cBRS) was different between women and men during eucapnia, isocapnic hypoxia, hypercapnic hyperoxia, and hypercapnic hypoxia. | cBRS was not different between women and men. | cBRS in women and men during eucapnia, isocapnic hypoxia, hypercapnic hyperoxia, and hypercapnic hypoxia. | Same as above | Same as above | Same as above | **cBRS:**  **in gain in women vs. men:**  **P=0.763**  **in number of sequences in women vs. men:**  **P=0.154**  **in BEI in women vs. men:**  **P=0.503** | Same as above | Same as above | Women vs Men | Same as above |  |  |
| Heart rate variability (HRV) indices were different between eucapnia, isocapnic hypoxia, hypercapnic hyperoxia, and hypercapnic hypoxia. | The square root of the mean of the sum of successive differences in R–R interval (RMSSD), the standard deviation of all normal sinus R–R intervals (SDNN), high frequency (HF in m^2^ and n.u.), and total power were reduced during isocapnic hypoxia and hypercapnic hypoxia compared to eucapnia.  Low frequency (LF in m^2^ and n.u.) was lower during all trials versus eucapnia. | HRV during eucapnia, isocapnic hypoxia, hypercapnic hyperoxia, and hypercapnic hypoxia. | **HRV in:**  **RMSSD during:**  - Eucapnia Women: 73.1±41.2 ms vs. Men: 69.2±34.9 ms  - Isocapnic hypoxia Women: 39.1±15.8 ms vs. Men: 51.5±24.3 ms  - Hypercapnic hyperoxia Women: 82.5±39.3 ms vs. Men: 69.1±44.3 ms  - Hypercapnic hypoxia Women: 51.0±31.0 ms vs. Men: 40.6±25.7 ms  **SDNN during:**  - Eucapnia Women: 77.8±31.4 ms vs. Men: 76.2±31.3 ms  - Isocapnic hypoxia Women: 54.5±21.0 ms vs. Men: 60.7±21.1 ms  - Hypercapnic hyperoxia Women: 76.4±27.6 ms vs. Men: 62.1±23.6 ms  - Hypercapnic hypoxia Women: 58.6±21.5 ms vs. Men: 46.1±19.8 ms  **HF during:**  - Eucapnia Women: 3223±4205 ms^2^ vs. Men: 2560±2170 ms^2^  - Isocapnic hypoxia Women: 703±456 ms^2^ vs. Men: 1653±1739 ms^2^  - Hypercapnic hyperoxia Women: 3457±2867 ms^2^ vs. Men: 1785±1520 ms^2^  - Hypercapnic hypoxia Women: 1425±1761 ms^2^ vs. Men: 741±848 ms^2^  **LF during:**  - Eucapnia Women: 2159±1948 ms^2^ vs. Men: 2057±1909 ms^2^  - Isocapnic hypoxia Women: 790±1520 ms^2^ vs. Men: 1061±1334 ms^2^  - Hypercapnic hyperoxia Women: 866±1364 ms^2^ vs. Men: 709±497 ms^2^  - Hypercapnic hypoxia Women: 480±772 ms^2^ vs. Men: 443±522 ms^2^  **Total Power during:**  - Eucapnia Women: 4874±4908 vs. Men: 4674±3971  - Isocapnic hypoxia Women: 1251±1769 vs. Men: 2115±4072  - Hypercapnic hyperoxia Women: 3014±3979 vs. Men: 2335±1840  - Hypercapnic hypoxia Women: 1175±2394 vs. Men: 990±1353  **HF (n.u.) during:**  - Eucapnia Women: 60±27 n.u. vs. Men: 58±22 n.u.  - Isocapnic hypoxia Women: 59±19 n.u. vs. Men: 61±21 n.u.  - Hypercapnic hyperoxia Women: 77±16 n.u. vs. Men: 66±18 n.u.  - Hypercapnic hypoxia Women: 62±21 n.u. vs. Men: 52±22 n.u.  **LF (n.u.) during:**  - Eucapnia Women: 40±27 n.u. vs. Men: 42±22 n.u.  - Isocapnic hypoxia Women: 41±19 n.u. vs. Men: 39±21 n.u.  - Hypercapnic hyperoxia Women: 23±16 n.u. vs. Men: 34±18 n.u.  - Hypercapnic hypoxia Women: 38±21 n.u. vs. Men: 48±22 n.u.  **LF/HF during:**  - Eucapnia Women: 2.62±4.78 vs. Men: 1.67±2.35  - Isocapnic hypoxia Women: 1.51±1.96 vs. Men: 1.22±1.53  - Hypercapnic hyperoxia Women: 0.49±0.59 vs. Men: 0.85±0.95  - Hypercapnic hypoxia Women: 1.40±1.69 vs. Men: 2.06±1.89 | Same as above | Same as above | **HRV in:**  **RMSSD:**  - Isocapnic hypoxia vs. eucapnia: **P=0.007**  -Hypercapnic hyperoxia vs. eucapnia: **P=1.000**  -Hypercapnic hypoxia vs. eucapnia: **P=0.008**  -Isocapnic hypoxia vs. hypercapnic hyperoxia: **P=0.001**  -Isocapnic hypoxia vs. hypercapnic hypoxia: **P=1.000**  **-**Hypercapnic hyperoxia vs. hypercapnic hypoxia: **P=0.001**  **SDNN:**  - Isocapnic hypoxia vs. eucapnia: **P=0.004**  -Hypercapnic hyperoxia vs. eucapnia: **P=0.916**  -Hypercapnic hypoxia vs. eucapnia: **P=0.001**  -Isocapnic hypoxia vs. hypercapnic hyperoxia: **P=0.205**  -Isocapnic hypoxia vs. hypercapnic hypoxia: **P=1.000**  **-**Hypercapnic hyperoxia vs. hypercapnic hypoxia: **P=0.016**  **HF:**  - Isocapnic hypoxia vs. eucapnia: **P=0.046**  -Hypercapnic hyperoxia vs. eucapnia: **P=1.000**  -Hypercapnic hypoxia vs. eucapnia: **P=0.045**  -Isocapnic hypoxia vs. hypercapnic hyperoxia: **P=0.186**  -Isocapnic hypoxia vs. hypercapnic hypoxia: **P=1.000**  **-**Hypercapnic hyperoxia vs. hypercapnic hypoxia: **P=0.131**  **LF:**  - Isocapnic hypoxia vs. eucapnia: **P=0.004**  -Hypercapnic hyperoxia vs. eucapnia: **P=0.001**  -Hypercapnic hypoxia vs. eucapnia: **P<0.0001**  -Isocapnic hypoxia vs. hypercapnic hyperoxia: **P=1.000**  -Isocapnic hypoxia vs. hypercapnic hypoxia: **P=0.984**  **-**Hypercapnic hyperoxia vs. hypercapnic hypoxia: **P=1.000**  **Total Power:**  - Isocapnic hypoxia vs. eucapnia: **P=0.004**  -Hypercapnic hyperoxia vs. eucapnia: **P=0.171**  -Hypercapnic hypoxia vs. eucapnia: **P<0.0001**  -Isocapnic hypoxia vs. hypercapnic hyperoxia: **P=1.000**  -Isocapnic hypoxia vs. hypercapnic hypoxia: **P=1.000**  **-**Hypercapnic hyperoxia vs. hypercapnic hypoxia: **P=0.131**  **HF (n.u.):**  **P=0.061**  **LF (n.u.):**  **P=0.061**  **LF/HF:**  **P=0.209** | Table 2 | HRV in RMSSD = ms  HRV in SDNN = ms  HRV in HF = ms^2^ and n.u.  HRV in LF = ms^2^ and n.u.  HRV in total power = ms^2^ | Eucapnia vs. isocapnic hypoxia vs. hypercapnic hyperoxia vs. hypercapnic hypoxia | Same as above |  |  |
| HRV indices were different between women and men during eucapnia, isocapnic hypoxia, hypercapnic hyperoxia, and hypercapnic hypoxia. | HRV indices were not different between women and men. | HRV in women and men during eucapnia, isocapnic hypoxia, hypercapnic hyperoxia, and hypercapnic hypoxia. | Same as above | Same as above | Same as above | **HRV in:**  **RMSSD in women vs. men:**  **P=0.744**  **SDNN in women vs. men:**  **P=0.549**  **HF in women vs. men:**  **P=0.403**  **LF in women vs. men:**  **P=0.986**  **Total Power in women vs. men:**  **P=0.738**  **HF (n.u.) in women vs. men:**  **P=0.403**  **LF (n.u.) in women vs. men:**  **P=0.403**  **LF/HF in women vs. men:**  **P=0.923** | Same as above | Same as above | Women vs Men | Same as above |  |  |
| Sympathetic neurovascular transduction was different between eucapnia, isocapnic hypoxia, hypercapnic hyperoxia, and hypercapnic hypoxia. | Sympathetic neurovascular transduction, assessed from the quotient of TPRi and total MSNA (TPRi/total MSNA), was similarly decreased during hypercapnic hyperoxia and hypercapnic hypoxia compared to eucapnia and isocapnic hypoxia. | TPRi/total MSNA during eucapnia, isocapnic hypoxia, hypercapnic hyperoxia, and hypercapnic hypoxia. | **TPRi/total MSNA during:**  - Eucapnia Women: 0.04±0.02 a.u. vs. Men: 0.03±0.02 a.u.  - Isocapnic hypoxia Women: 0.02±0.01 a.u. vs. Men: 0.02±0.008 a.u.  - Hypercapnic hyperoxia Women: 0.01±0.007 a.u. vs. Men: 0.01±0.006 a.u.  - Hypercapnic hypoxia Women: 0.007±0.004 a.u. vs. Men: 0.01±0.006 a.u. | Same as above | Women: n=10; Men: n=10 | **TPRi/total MSNA:**  - Isocapnic hypoxia vs. eucapnia: **P<0.0001**  -Hypercapnic hyperoxia vs. eucapnia: **P<0.0001**  -Hypercapnic hypoxia vs. eucapnia: **P<0.0001**  -Isocapnic hypoxia vs. hypercapnic hyperoxia: **P=0.100**  -Isocapnic hypoxia vs. hypercapnic hypoxia: **P=0.004**  **-**Hypercapnic hyperoxia vs. hypercapnic hypoxia: **P=1.000** | Figure 5 | TPRi/total MSNA = a.u. | Eucapnia vs. isocapnic hypoxia vs. hypercapnic hyperoxia vs. hypercapnic hypoxia | Same as above |  |  |
| Sympathetic neurovascular transduction was different between women and men during eucapnia, isocapnic hypoxia, hypercapnic hyperoxia, and hypercapnic hypoxia. | Sympathetic neurovascular transduction, assessed from the quotient of TPRi and total MSNA (TPRi/total MSNA), was not different between women and men. | TPRi/total MSNA in women and men during eucapnia, isocapnic hypoxia, hypercapnic hyperoxia, and hypercapnic hypoxia. | Same as above | Same as above | Same as above | **TPRi/total MSNA in women vs. men:**  **P=0.504** | Same as above | Same as above | Women vs Men | Same as above |  |  |
| Sympathetic neurovascular transduction was different between eucapnia, isocapnic hypoxia, hypercapnic hyperoxia, and hypercapnic hypoxia | Sympathetic neurovascular transduction, assessed from the magnitude of the peak rise in DBP following multiple MSNA bursts, was blunted during isocapnic hypoxia, hypercapnic hyperoxia, and hypercapnic hypoxia compared to eucapnia | Peak rise in DBP following single or multiple MSNA bursts during eucapnia, isocapnic hypoxia, hypercapnic hyperoxia, and hypercapnic hypoxia | **Peak rise in DBP following:**  - **Single MSNA burst** **during:**  - Eucapnia Women: 3.51±1.45 mmHg vs. Men: 2.60±1.78 mmHg  - Isocapnic hypoxia Women: 1.94±0.80 mmHg vs. Men: 2.33±1.69 mmHg  - Hypercapnic hyperoxia Women: 2.44±1.50 mmHg vs. Men: 1.80±1.49 mmHg  - Hypercapnic hypoxia Women: 2.64±2.62 mmHg vs. Men: 2.80±2.09 mmHg  - **Multiple MSNA burst** **during:**  - Eucapnia Women: 6.32±2.51 mmHg vs. Men: 6.58±2.78 mmHg  - Isocapnic hypoxia Women: 3.87±1.88 mmHg vs. Men: 4.42±2.00 mmHg  - Hypercapnic hyperoxia Women: 3.83±2.32 mmHg vs. Men: 4.96±1.78 mmHg  - Hypercapnic hypoxia Women: 4.73±2.79 mmHg vs. Men: 4.59±1.61 mmHg | Same as above | Same as above | **Peak rise in DBP following:**  **Single MSNA burst**  **P=0.126**  **Multiple MSNA burst**  -Isocapnic hypoxia vs. eucapnia: **P=0.003**  -Hypercapnic hyperoxia vs. eucapnia: **P=0.011**  -Hypercapnic hypoxia vs. eucapnia: **P=0.037**  -Isocapnic hypoxia vs. hypercapnic hyperoxia: **P=1.000**  -Isocapnic hypoxia vs. hypercapnic hypoxia: **P=1.000**  **-**Hypercapnic hyperoxia vs. hypercapnic hypoxia: **P=1.000** | Same as above | Peak rise in DBP = mmHg | Eucapnia vs. isocapnic hypoxia vs. hypercapnic hyperoxia vs. hypercapnic hypoxia | Same as above |  |  |
| Sympathetic neurovascular transduction was different between women and men during eucapnia, isocapnic hypoxia, hypercapnic hyperoxia, and hypercapnic hypoxia. | Sympathetic neurovascular transduction, assessed from the magnitude of the peak rise in DBP following multiple MSNA bursts, was not different between women and men. | Peak rise in DBP following single or multiple MSNA bursts in women and men during eucapnia, isocapnic hypoxia, hypercapnic hyperoxia, and hypercapnic hypoxia. | Same as above | Same as above | Same as above | **Single MSNA burst in women vs. men:**  **P=0.650**  **Multiple MSNA bursts in women vs. men:**  **P=0.497** | Same as above | Same as above | Women vs Men | Same as above |  |  |
| Peak change in diastolic blood pressure (DBP) following a cardiac cycle both with and without spontaneous muscle sympathetic nerve activity (MSNA) bursts were different between eucapnia, isocapnic hypoxia, hypercapnic hyperoxia, and hypercapnic hypoxia. | The magnitude of the peak DBP following a cardiac cycle with MSNA bursts was attenuated only during hypercapnic hyperoxia compared to eucapnia. | The magnitude of the peak DBP following a cardiac cycle with or without MSNA bursts during eucapnia, isocapnic hypoxia, hypercapnic hyperoxia, and hypercapnic hypoxia. | **Peak DBP following:**  - **A cardiac cycle with MSNA burst** **during**:  - Eucapnia Women: 5.4±1.1 mmHg vs. Men: 5.7±1.3 mmHg  - Isocapnic hypoxia Women: 6.0±1.4 mmHg vs. Men: 6.3±1.9 mmHg  - Hypercapnic hyperoxia Women: 4.4±1.3 mmHg vs. Men: 5.5±1.7 mmHg  - Hypercapnic hypoxia Women: 6.2±1.7 mmHg vs. Men: 6.1±2.3 mmHg  - **A cardiac cycle without MSNA bursts** **during**:  - Eucapnia Women: 5.3±0.9 mmHg vs. Men: 5.0±2.4 mmHg  - Isocapnic hypoxia Women: 5.6±1.6 mmHg vs. Men: 6.2±1.5 mmHg  - Hypercapnic hyperoxia Women: 4.7±1.3 mmHg vs. Men: 3.7±1.8 mmHg  - Hypercapnic hypoxia Women: 5.5±2.2 mmHg vs. Men: 5.9±1.8 mmHg | Same as above | Same as above | **Peak DBP following:**  **A cardiac cycle with MSNA burst** **during**:  -Isocapnic hypoxia vs. eucapnia: **P=0.298**  -Hypercapnic hyperoxia vs. eucapnia: **P=0.033**  -Hypercapnic hypoxia vs. eucapnia: **P=0.059**  -Isocapnic hypoxia vs. hypercapnic hyperoxia: **P=1.000**  -Isocapnic hypoxia vs. hypercapnic hypoxia: **P=1.000**  **-**Hypercapnic hyperoxia vs. hypercapnic hypoxia: **P=1.000**  **A cardiac cycle without MSNA bursts:**  **P=0.161** | Table 3 | Peak DBP following a cardiac cycle with MSNA burst = mmHg  Peak DBP following a cardiac cycle without MSNA burst = mmHg | Eucapnia vs. isocapnic hypoxia vs. hypercapnic hyperoxia vs. hypercapnic hypoxia | Same as above |  |  |
| The magnitude of the peak DBP following a cardiac cycle with or without MSNA bursts was different between women and men during eucapnia, isocapnic hypoxia, hypercapnic hyperoxia, and hypercapnic hypoxia. | The magnitude of the peak DBP following a cardiac cycle with or without MSNA bursts was not different between women and men. | The magnitude of the peak DBP following a cardiac cycle with or without MSNA bursts in women and men during eucapnia, isocapnic hypoxia, hypercapnic hyperoxia, and hypercapnic hypoxia. | Same as above | Same as above | Same as above | **Peak DBP following:**  **A cardiac cycle with MSNA bursts** **in women vs. men:**  **P=0.819**  - **A cardiac cycle without MSNA bursts** **in women vs. men:**  **P=0.682** | Same as above | Same as above | Women vs Men | Same as above |  |  |
| Partial pressure of end-tidal oxygen (P_ET_O_2_) was different similar between isocapnic hyperoxia, and hypocapnic hyperoxia, but elevated compared to eucapnia. | P_ET_O_2_ was similarly elevated during isocapnic hyperoxia and hypocapnic hyperoxia compared to eucapnia. | P_ET_O_2_ during eucapnia, isocapnic hyperoxia, and hypocapnic hyperoxia. | **P_ET_O_2_ during:**  - Eucapnia Women: 97±5 mmHg vs. Men: 96±9 mmHg  - Isocapnic hyperoxia Women: 300±28 mmHg vs. Men: 316±31 mmHg  - Hypocapnic hyperoxia Women: 320±41 mmHg vs. Men: 320±29 mmHg | Same as above | Same as above | **P_ET_O_2_ during:**  -Isocapnic hyperoxia vs. eucapnia: **P<0.0001**  -Hypocapnic hyperoxia vs. eucapnia: **P<0.0001**  -Isocapnic hyperoxia vs. hypocapnic hyperoxia: **P=0.313** | Table 4 | mmHg | Eucapnia vs. isocapnic hyperoxia vs. hypocapnic hyperoxia | Same as above |  |  |
| Partial pressure of end-tidal oxygen (P_ET_O_2_) was similar between women and men during eucapnia, isocapnic hyperoxia, and hypocapnic hyperoxia. | There were no sex-differences in the P_ET_O_2_ during eucapnia, isocapnic hyperoxia, and hypocapnic hyperoxia. | P_ET_O_2_ in women and men during eucapnia, isocapnic hyperoxia, and hypocapnic hyperoxia. | Same as above | Same as above | Same as above | **P_ET_O_2_ in women vs. men:** **P=0.572** | Same as above | Same as above | Women vs. Men | Same as above |  |  |
| Partial pressure of end-tidal carbon dioxide (P_ET_CO_2_) was similar between eucapnia and isocapnic hyperoxia, but different from hypocapnic hyperoxia. | P_ET_CO_2_ was similarly reduced during hypocapnic hyperoxia compared to eucapnia and isocapnic hyperoxia. | P_ET_CO_2_ during eucapnia, isocapnic hyperoxia, and hypocapnic hyperoxia | **P_ET_CO_2_ during:**  - Eucapnia Women: 41±2 mmHg vs. Men: 42±1 mmHg  - Isocapnic hyperoxia Women: 41±4 mmHg vs. Men: 42±3 mmHg  - Hypocapnic hyperoxia Women: 36±5 mmHg vs. Men: 37±4 mmHg | Same as above | Same as above | **P_ET_CO_2_ during:**  -Isocapnic hyperoxia vs. eucapnia: **P=0.963**  -Hypocapnic hyperoxia vs. eucapnia: **P<0.0001**  -Isocapnic hyperoxia vs. hypocapnic hyperoxia: **P=0.002** | Table 4 | mmHg | Eucapnia vs. isocapnic hyperoxia vs. hypocapnic hyperoxia | Same as above |  |  |
| Partial pressure of end-tidal carbon dioxide (P_ET_CO_2_) was similar between women and men during eucapnia, isocapnic hyperoxia, and hypocapnic hyperoxia. | There were no sex-differences in the P_ET_CO_2_ during eucapnia, isocapnic hyperoxia, and hypocapnic hyperoxia. | P_ET_CO_2_ in women and men during eucapnia, isocapnic hyperoxia, and hypocapnic hyperoxia. | Same as above | Same as above | Same as above | **P_ET_CO_2_ in women vs. men:** **P=0.131** | Same as above | Same as above | Women vs. Men | Same as above |  |  |
| Oxygen saturation (S_P_O_2_) was different between eucapnia, isocapnic hyperoxia, and hypocapnic hyperoxia. | S_P_O_2_ was similarly elevated during isocapnic hyperoxia and hypocapnic hyperoxia compared to eucapnia. | S_P_O_2_ during eucapnia, isocapnic hyperoxia, and hypocapnic hyperoxia. | **S_P_O_2_ during:**  - Eucapnia Women: 97±1 % vs. Men: 97±1 %  - Isocapnic hyperoxia Women: 99±1 % vs. Men: 99±0 %  - Hypocapnic hyperoxia Women: 99±1 % vs. Men: 99±0 % | Same as above | Same as above | **S_P_O_2_ during:**  -Isocapnic hyperoxia vs. eucapnia: **P<0.0001**  -Hypocapnic hyperoxia vs. eucapnia: **P<0.0001**  -Isocapnic hyperoxia vs. hypocapnic hyperoxia: **P=0.759** | Table 4 | % | Eucapnia vs. isocapnic hyperoxia vs. hypocapnic hyperoxia | Same as above |  |  |
| Oxygen saturation (S_P_O_2_) was similar between women and men during eucapnia, isocapnic hyperoxia, and hypocapnic hyperoxia. | There were no sex-differences in the S_P_O_2_ during eucapnia, isocapnic hyperoxia, and hypocapnic hyperoxia. | S_P_O_2_ in women and men during eucapnia, isocapnic hyperoxia, and hypocapnic hyperoxia. | Same as above | Same as above | Same as above | **S_P_O_2_ in women vs. men:**  **P=0.960** | Same as above | Same as above | Women vs. Men | Same as above |  |  |
| Respiratory variables (ventilation, V_E_; tidal volume, V_T_; and respiratory frequency, R*f*) were different between eucapnia, isocapnic hyperoxia, and hypocapnic hyperoxia. | Hypocapnic hyperoxia increased V̇_E_ and R*f* compared to eucapnia and isocapnic hyperoxia. | V̇_E_, V̇_T_, and R*f* during eucapnia, isocapnic hyperoxia, and hypocapnic hyperoxia. | **V̇_E_ during:**  - Eucapnia Women: 12.1±1.9 L·min^-1^ vs. Men: 13.9±4.0 L·min^-1^  - Isocapnic hyperoxia Women: 14.2±4.2 L·min^-1^ vs. Men: 14.4±3.9 L·min^-1^  - Hypocapnic hyperoxia Women: 18.1±4.0 L·min^-1^ vs. Men: 18.9±5.7 L·min^-1^  **V̇_T_ during:**  - Eucapnia Women: 0.93±0.27 L vs. Men: 0.85±0.22 L  - Isocapnic hyperoxia Women: 0.95±0.37 L vs. Men: 0.95±0.36 L  - Hypocapnic hyperoxia Women: 0.76±0.20 L vs. Men: 0.91±0.26 L  **R*f* during:**  - Eucapnia Women: 13±5 breaths·min^-1^ vs. Men: 15±4 breaths·min^-1^  - Isocapnic hyperoxia Women: 15±4 breaths·min^-1^ vs. Men: 15±5 breaths·min^-1^  - Hypocapnic hyperoxia Women: 23±7 breaths·min^-1^ vs. Men: 19±5 breaths·min^-1^ | Same as above | Same as above | **V̇_E_ during:**  -Isocapnic hyperoxia vs. eucapnia: **P=0.705**  -Hypocapnic hyperoxia vs. eucapnia: **P<0.0001**  -Isocapnic hyperoxia vs. hypocapnic hyperoxia: **P=0.002**  **V̇_T_**  **P=0.209**  **R*f* during:**  -Isocapnic hyperoxia vs. eucapnia: **P=1.000**  -Hypocapnic hyperoxia vs. eucapnia: **P<0.0001**  -Isocapnic hyperoxia vs. hypocapnic hyperoxia: **P<** **0.0001** | Table 4 | V̇_E_ = L·min^-1^  V̇_T_ = L  R*f* = breaths·min^-1^ | Eucapnia vs. isocapnic hyperoxia vs. hypocapnic hyperoxia. | Same as above |  |  |
| V̇_E_, V̇_T_, and R*f* were different between women and men during eucapnia, isocapnic hyperoxia, and hypocapnic hyperoxia. | V̇_E_ and V̇_T_ were not different in women and men during eucapnia, isocapnic hyperoxia, and hypocapnic hyperoxia. | V̇_E_, V̇_T_, and R*f* in women and men during eucapnia, isocapnic hyperoxia, and hypocapnic hyperoxia. | Same as above | Same as above | Same as above | **V̇_E_ in women vs. men:**  **P=0.515**  **V̇_T_ in women vs. men:**  **P=0.803**  **R*f* in women vs. men: P=0.671** | Same as above | Same as above | Women vs Men | Same as above |  |  |
| Perception of breathlessness was different between eucapnia, isocapnic hyperoxia, and hypocapnic hyperoxia. | There was no difference in the perception of breathlessness during eucapnia, isocapnic hyperoxia, and hypocapnic hyperoxia. | Perception of breathlessness during eucapnia, isocapnic hyperoxia, and hypocapnic hyperoxia. | **Perception of breathlessness during:**  - Eucapnia Women: 0±0 a.u. vs. Men: 0±0 a.u.  - Isocapnic hyperoxia Women: 1±2 a.u. vs. Men: 0±0 a.u.  - Hypocapnia hyperoxia Women: 1±1 a.u. vs. Men: 1±0 a.u. | Same as above | Same as above | **Perception of breathlessness:**  **P=0.052** | Table 4 | a.u. | Eucapnia vs. isocapnic hyperoxia vs. hypocapnic hyperoxia | Same as above |  |  |
| Perception of breathlessness was similar between women and men during eucapnia, isocapnic hyperoxia, and hypocapnic hyperoxia. | There were no sex-differences in the perception of breathlessness during eucapnia, isocapnic hyperoxia, and hypocapnic hyperoxia. | Perception of breathlessness in women and men during eucapnia, isocapnic hyperoxia, and hypocapnic hyperoxia. | Same as above | Same as above | Same as above | **Perception of breathlessness in women vs. men:**  **P=0.512** | Same as above | Same as above | Women vs. Men | Same as above |  |  |
| Cardiovascular variables (heart rate, HR; systolic blood pressure, SBP; diastolic blood pressure, DBP; mean arterial pressure, MAP; cardiac output, CO; stroke volume, SV; and total peripheral resistance, TPR) were different between eucapnia, isocapnic hyperoxia, and hypocapnic hyperoxia. | Isocapnic hyperoxia and hypocapnic hyperoxia similarly increased MAP and TPR compared to eucapnia.  Hypocapnic hyperoxia increased DBP. | HR, SBP, DBP, MAP, CO, SV, and TPR during eucapnia, isocapnic hyperoxia, and hypocapnic hyperoxia. | **HR during:**  - Eucapnia Women: 70±10 beats·min-^1^ vs. Men: 69±7 beats·min-^1^  - Isocapnic hyperoxia Women: 65±8 beats·min-^1^ vs. Men: 64±5 beats·min-^1^  - Hypocapnic hyperoxia Women: 69±8 beats·min-^1^ vs. Men: 67±6 beats·min-^1^  **SBP during:**  - Eucapnia Women: 113±10 mmHg vs. Men: 126±10 mmHg  - Isocapnic hyperoxia Women: 114±11 mmHg vs. Men: 131±9 mmHg  - Hypocapnic hyperoxia Women: 121±7 mmHg vs. Men: 130±10 mmHg  **DBP during:**  - Eucapnia Women: 71±7 mmHg vs. Men: 68±12 mmHg  - Isocapnic hyperoxia Women: 72±9 mmHg vs. Men: 75±12 mmHg  - Hypocapnic hyperoxia Women: 76±11 mmHg vs. Men: 78±9 mmHg  **MAP during:**  - Eucapnia Women: 85±7 mmHg vs. Men: 88±12 mmHg  - Isocapnic hyperoxia Women: 87±8 mmHg vs. Men: 97±13 mmHg  - Hypocapnia hyperoxia Women: 92±12 mmHg vs. Men: 98±11 mmHg  **CO during:**  - Eucapnia Women: 5.6±1.7 L·min^-1^ vs. Men: 5.9±1.2 L·min^-1^  - Isocapnic hyperoxia Women: 5.2±1.3 L·min^-1^ vs. Men: 5.7±1.3 L·min^-1^  - Hypocapnic hyperoxia Women: 5.5±1.7 L·min^-1^ vs. Men: 5.9±1.4 L·min^-1^  **SV during:**  - Eucapnia Women: 84±20 mL vs. Men: 87±18 mL  - Isocapnic hyperoxia Women: 84±21 mL vs. Men: 90±23 mL  - Hypocapnic hyperoxia Women: 83±25 mL vs. Men: 88±21 mL  **TPR during:**  - Eucapnia Women: 16.5±5.4 mmHg·L^-1^·min^-1^ vs. Men: 15.6±4.5 mmHg·L^-1^·min^-1^  - Isocapnic hyperoxia Women: 17.6±4.6 mmHg·L^-1^·min^-1^ vs. Men: 18.3±6.5 mmHg·L^-1^·min^-1^  - Hypocapnic hyperoxia Women: 18.3±6.6 mmHg·L^-1^·min^-1^ vs. Men: 17.9±5.7 mmHg·L^-1^·min^-1^ | Same as above | Same as above | **HR during:**  -Isocapnic hyperoxia vs. eucapnia: **P=0.002**  -Hypocapnic hyperoxia vs. eucapnia: **P=1.000**  -Isocapnic hyperoxia vs. hypocapnic hyperoxia: **P=0.016**  **SBP:**  **P=0.068**  **DBP during:**  -Isocapnic hyperoxia vs. eucapnia: **P=0.151**  -Hypocapnic hyperoxia vs. eucapnia: **P=0.001**  -Isocapnic hyperoxia vs. hypocapnic hyperoxia: **P=0.194**  **MAP during:**  -Isocapnic hyperoxia vs. eucapnia: **P=0.031**  -Hypocapnic hyperoxia vs. eucapnia: **P<0.0001**  -Isocapnic hyperoxia vs. hypocapnic hyperoxia: **P=0.399**  **CO:**  **P=0.143**  **SV**  **P=0.563**  **- TPR during:**  -Isocapnic hyperoxia vs. eucapnia: **P=0.004**  -Hypocapnic hyperoxia vs. eucapnia: **P=0.002**  -Isocapnic hyperoxia vs. hypocapnic hyperoxia: **P=1.000** | Table 4 | HR = beats·min^-1^  SBP = mmHg  DBP = mmHg  MAP = mmHg  CO = L·min^-1^  SV = mL  TPR = mmHg·L^-1^·min^-1^ | Eucapnia vs. isocapnic hyperoxia vs. hypocapnic hyperoxia | Same as above |  |  |
| Cardiovascular variables (heart rate, HR; systolic blood pressure, SBP; diastolic blood pressure, DBP; mean arterial pressure, MAP; cardiac output, CO; stroke volume, SV; and total peripheral resistance, TPR) were different between women and men during eucapnia, isocapnic hyperoxia, and hypocapnic hyperoxia. | Overall, SBP was lower in women, while no other sex-differences were evident. | HR, SBP, DBP, MAP, CO, SV, and TPR in women and men during eucapnia, isocapnic hyperoxia, and hypocapnic hyperoxia. | Same as above | Same as above | Same as above | **HR in women vs. men:** **P=0.666**  **SBP in women vs. men during:**  **-** Eucapnia **P=** **0.011**  - Isocapnic hyperoxia **P=** **0.010**  - Hypocapnic hyperoxia **P=0.018**  **DBP in women vs. men:** **P=0.858**  **MAP in women vs. men:** **P=0.149**  **CO in women vs. men:** **P=0.550**  **SV in women vs. men:** **P=0.585**  **TPR in women vs. men:** **P=0.932** | Same as above | Same as above | Women vs. Men | Same as above |  |  |
| Muscle sympathetic nerve activity (MSNA) in burst frequency (BF), burst incidence (BI), burst amplitude, and total MSNA were different between eucapnia, isocapnic hyperoxia, and hypercapnic hyperoxia. | MSNA amplitude was increased during isocapnic hyperoxia, compared to eucapnia. | MSNA during eucapnia, isocapnic hyperoxia, and hypercapnic hyperoxia. | **MSNA in BF during:**  - Eucapnia Women: 11±3 bursts·min^-1^ vs. Men: 11±5 bursts ·min^-1^  - Isocapnic hyperoxia Women: 11±4 bursts·min^-1^ vs. Men: 13±5 bursts ·min^-1^  - Hypocapnic hyperoxia Women: Women: 12±4 bursts·min^-1^ vs. Men: 12±4 bursts ·min^-1^  **MSNA in BI during:**  - Eucapnia Women: 16±2 bursts·100 heartbeats^-1^ vs. Men: 17±2 bursts ·100 heartbeats^-1^  - Isocapnic hyperoxia Women: 17±7 bursts·100 heartbeats^-1^ vs. Men: 20±8 bursts ·100 heartbeats^-1^  - Hypocapnic hyperoxia Women: 17±6 bursts·100 heartbeats^-1^ vs. Men: 18±7 bursts ·100 heartbeats^-1^  **MSNA in burst amplitude during:**  - Eucapnia Women: 100±0 % vs. Men: 100±0 %  - Isocapnic hyperoxia Women: 133±21 % vs. Men: 106±27 %  - Hypocapnic hyperoxia Women: 123±29 % vs. Men: 114±37 %  **Total MSNA during:**  - Eucapnia Women: 100±0 % vs. Men: 100±0 %  - Isocapnic hyperoxia Women: 100±27 % vs. Men: 102±53 %  - Hypocapnic hyperoxia Women: 95±34 % vs. Men: 90±31 % | Same as above | Same as above | **MSNA in BF:**  **P=0.722**  **MSNA in BI:**  **P=0.225**  **MSNA in burst amplitude:**  -Isocapnic hyperoxia vs. eucapnia: **P=0.047**  -Hypocapnic hyperoxia vs. eucapnia: **P=0.056**  -Isocapnic hyperoxia vs. hypocapnic hyperoxia: **P=1.000**  **Total MSNA:**  **P=0.644** | Table 4 | MSNA in BF = bursts·min^-1^  MSNA in BI = bursts·100 heartbeats^-1^  MSNA in burst amplitude = %  Total MSNA = % | Eucapnia vs. isocapnic hyperoxia vs. hypocapnic hyperoxia | Same as above |  |  |
| Muscle sympathetic nerve activity (MSNA) in burst frequency (BF), burst incidence (BI), burst amplitude, and total MSNA were different between women and men during eucapnia, isocapnic hyperoxia, and hypocapnic hyperoxia. | There were no sex-differences in MSNA during eucapnia, isocapnic hyperoxia, and hypocapnic hyperoxia. | MSNA in women and men during eucapnia, isocapnic hyperoxia, and hypocapnic hyperoxia. | Same as above | Same as above | Same as above | **MSNA in BF in women vs. men:**  **P=0.693**  **MSNA in BI in women vs. men:**  **P=0.694**  **MSNA in burst amplitude in women vs. men:**  **P=0.063**  **Total MSNA in women vs. men:**  **P=0.893** | Same as above | Same as above | Women vs Men | Same as above |  |  |
| Baroreflex sensitivity was different between eucapnia, isocapnic hyperoxia, and hypocapnic hyperoxia. | There were no differences in arterial baroreflex control of MSNA (ABR-MSNA) between eucapnia, isocapnic hyperoxia, and hypocapnic hyperoxia. | ABR-MSNA during eucapnia, isocapnic hyperoxia, and hypocapnic hyperoxia. | **ABR-MSNA in BI during:**  - Eucapnia Women: -4.14±1.45 bursts [100 heartbeats]^-1^·mmHg^-1^ vs. Men: -3.80±1.54 bursts [100 heartbeats]^-1^·mmHg^-1^  - Isocapnic hyperoxia Women: -4.35±1.87 bursts [100 heartbeats]^-1^·mmHg^-1^ vs. Men: -4.22±2.53 bursts [10 heartbeats]^-1^·mmHg^-1^  - Hypocapnic hyperoxia Women: -4.29±2.63 bursts [100 heartbeats]^-1^·mmHg^-1^ vs. Men: -3.98±1.48 bursts [100 heartbeats]^-1^·mmHg^-1^  **ABR-MSNA in Total MSNA during:**  - Eucapnia Women: -2.50±0.81 a.u.·beat^-1^·mmHg^-1^ vs. Men: -2.21±1.00 a.u.·beat^-1^·mmHg^-1^  - Isocapnic hyperoxia Women: -2.92±1.45 a.u.·beat^-1^·mmHg^-1^ vs. Men: -2.68±1.58 a.u.·beat^-1^·mmHg^-1^  - Hypocapnic hyperoxia Women: -2.67±1.47 a.u.·beat^-1^·mmHg^-1^ vs. Men: -2.47±0.98 a.u.·beat^-1^·mmHg^-1^ | Same as above | Women: n=9; Men: n=9 | **ABR-MSNA**  **in BI:** **P=0.751**  **in Total MSNA:**  **P=0.335** | Table 5 | ABR-MSNA in burst incidence = bursts [100 heartbeats]^-1^·mmHg^-1^  ABR-MSNA in Total MSNA = a.u.·beat^-1^·mmHg^-1^ | Eucapnia vs. isocapnic hyperoxia vs. hypocapnic hyperoxia | Same as above |  |  |
| Baroreflex sensitivity was different between women and men during eucapnia, isocapnic hyperoxia, and hypocapnic hyperoxia. | There were no sex-differences in ABR-MSNA during eucapnia, isocapnic hyperoxia, and hypocapnic hyperoxia. | ABR-MSNA in women and men during eucapnia, isocapnic hyperoxia, and hypocapnic hyperoxia. | Same as above | Same as above | Same as above | **ABR-MSNA:**  **in BI in women vs. men:**  **P=0.669**  **in total MSNA in women vs. men:**  **P=0.548** | Same as above | Same as above | Women vs Men | Same as above |  |  |
| Spontaneous cardiac baroreflex sensitivity (cBRS) was different between eucapnia, isocapnic hyperoxia, and hypocapnic hyperoxia. | Baroreflex sequences and BEI were decreased during hypocapnic hyperoxia compared to eucapnia.  Baroreflex gain was decreased during hypocapnic hyperoxia compared to isocapnic hyperoxia. | cBRS during eucapnia, isocapnic hyperoxia, and hypocapnic hyperoxia. | **cBRS in:**  **gain during:**  - Eucapnia Women: 15.6±5.0 ms·mmHg^-1^ vs. Men: 14.2±5.2 ms·mmHg^-1^  - Isocapnic hyperoxia Women: 18.1±5.5 ms·mmHg^-1^ vs. Men: 20.3±10.6 ms·mmHg^-1^  - Hypocapnic hyperoxia Women: 8.5±2.9 ms·mmHg^-1^ vs. Men: 15.0±10.8 ms·mmHg^-1^  **number of sequences during:**  - Eucapnia Women: 33±20 n vs. Men: 23±17 n  - Isocapnic hyperoxia Women: 18±16 n vs. Men: 16±15 a.u.·beat^-1^·mmHg^-1^  - Hypocapnic hyperoxia Women: 3±3 n vs. Men: 12±12 n  **BEI during:**  - Eucapnia Women: 0.57±0.32 % vs. Men: 0.50±0.25 %  - Isocapnic hyperoxia Women: 0.35±0.27 % vs. Men: 0.35±0.28 %  - Hypocapnic hyperoxia Women: 0.13±0.10 % vs. Men: 0.28±0.20 % | Same as above | Women: n=7; Men: n=8 | **cBRS in:**  **gain:**  - Isocapnic hyperoxia vs. eucapnia: **P=0.206**  -Hyorcapnic hyperoxia vs. eucapnia: **P=0.532**  -Isocapnic hyperoxia vs. hypocapnic hyperoxia: **P=0.009**  **number of sequences:**  - Isocapnic hyperoxia vs. eucapnia: **P=0.082**  -Hypocapnic hyperoxia vs. eucapnia: **P=0.001**  -Isocapnic hyperoxia vs. hypocapnic hyperoxia: **P=0.151**  **BEI:**  - Isocapnic hyperoxia vs. eucapnia: **P=0.086**  -Hypocapnic hyperoxia vs. eucapnia: **P=0.001**  -isocapnic hyperoxia vs. hypocapnic hyperoxia: **P=0.258** | Table 5 | cBRS in gain = ms·mmHg^-1^  cBRS in number of sequences = n  cBRS in BEI = % | Eucapnia vs. isocapnic hyperoxia vs. hypocapnic hyperoxia | Same as above |  |  |
| Spontaneous cardiac baroreflex sensitivity (cBRS) was different between women and men during eucapnia, isocapnic hyperoxia, and hypocapnic hyperoxia. | cBRS was not different between women and men. | cBRS in women and men during eucapnia, isocapnic hyperoxia, and hypocapnic hyperoxia. | Same as above | Same as above | Same as above | **cBRS in:**  **gain in women vs. men:**  **P=0.414**  **number of sequences in women vs. men:**  **P=0.884**  **BEI in women vs. men:**  **P=0.784** | Same as above | Same as above | Women vs Men | Same as above |  |  |
| Heart rate variability (HRV) indices were different between eucapnia, isocapnic hyperoxia, and hypocapnic hyperoxia. | SDNN, LF, and total power were decreased during hypocapnic hyperoxia compared to eucapnia.  RMSSD and HF were decreased during hypocapnic hyperoxia compared to isocapnic hyperoxia. | HRV during eucapnia, isocapnic hyperoxia, and hypocapnic hyperoxia. | **HRV in:**  **RMSSD during:**  - Eucapnia Women: 73.1±41.2 ms vs. Men: 69.2±34.9 ms  - Isocapnic hyperoxia Women: 82.7±49.9 ms vs. Men: 79.5±47.5 ms  - Hypocapnic hyperoxia Women: 55.0±31.4 ms vs. Men: 62.8±49.1 ms  **SDNN during:**  - Eucapnia Women: 77.8±31.4 ms vs. Men: 76.2±31.3 ms  - Isocapnic hyperoxia Women: 74.2±30.7 ms vs. Men: 83.8±38.8 ms  - Hypocapnic hyperoxia Women: 55.1±18.8 ms vs. Men: 63.1±31.4 ms  **HF during:**  - Eucapnia Women: 3223±4205 ms^2^ vs. Men: 2560±2170 ms^2^  - Isocapnic hyperoxia Women: 3651±4502 ms^2^ vs. Men: 3281±3539 ms^2^  - Hypocapnic hyperoxia Women: 1047±979 ms^2^ vs. Men: 1837±3137 ms^2^  **LF during:**  - Eucapnia Women: 2159±1948 ms^2^ vs. Men: 2057±1909 ms^2^  - Isocapnic hyperoxia Women: 1366±1585 ms^2^ vs. Men: 2610±3139 ms^2^  - Hypocapnic hyperoxia Women: 643±560 ms^2^ vs. Men: 988±921 ms^2^  **Total Power during:**  - Eucapnia Women: 4874±4908 vs. Men: 4674±3971  - Isocapnic hyperoxia Women: 3320±4859 vs. Men: 4548±6035  - Hypocapnic hyperoxia Women: 1535±1720 vs. Men: 2099±4006  **HF (n.u.) during:**  - Eucapnia Women: 60±27 n.u. vs. Men: 58±22 n.u.  - Isocapnic hyperoxia Women: 68±23 n.u. vs. Men: 56±23 n.u.  - Hypocapnic hyperoxia Women: 63±20 n.u. vs. Men: 56±25 n.u.  **LF during:**  - Eucapnia Women: 40±27 n.u. vs. Men: 42±22 n.u.  - Isocapnic hyperoxia Women: 30±22 n.u. vs. Men: 44±23 n.u.  - Hypocapnic hyperoxia Women: 36±20 n.u. vs. Men: 44±25 n.u.  **LF/HF during:**  - Eucapnia Women: 2.62±4.78 vs. Men: 1.67±2.35  - Isocapnic hyperoxia Women: 4.90±10.80 vs. Men: 1.97±2.27  - Hypocapnic hyperoxia Women: 1.25±1.90 vs. Men: 1.69±1.93 | Same as above | Same as above | **HRV in:**  **RMSSD:**  - Isocapnic hyperoxia vs. eucapnia: **P=0.3613**  -Hypocapnic hyperoxia vs. eucapnia: **P=0.172**  -Isocapnic hyperoxia vs. hypocapnic hyperoxia: **P=0.003**  **SDNN:**  - Isocapnic hyperoxia vs. eucapnia: **P=1.000**  -Hypocapnic hyperoxia vs. eucapnia: **P=0.0001**  -Isocapnic hyperoxia vs. hypocapnic hyperoxia: **P=0.0001**  **HF:**  - Isocapnic hyperoxia vs. eucapnia: **P=1.000**  -Hypocapnic hyperoxia vs. eucapnia: **P=0.097**  -Isocapnic hyperoxia vs. hypocapnic hyperoxia: **P=0.011**  **LF:**  - Isocapnic hyperoxia vs. eucapnia: **P=1.000**  -Hypocapnic hyperoxia vs. eucapnia: **P=0.007**  -Isocapnic hyperoxia vs. hypocapnic hyperoxia: **P=0.015**    **Total Power:**  - Isocapnic hyperoxia vs. eucapnia: **P=1.000**  -Hypocapnic hyperoxia vs. eucapnia: **P=0.003**  -Isocapnic hyperoxia vs. hypocapnic hyperoxia: **P=0.0001**  **HF (n.u.):**  **P=0.794**  **LF (n.u.):**  **P=0.691**  **LF/HF:**  **P=0.424** | Table 5 | HRV in RMSSD = ms  HRV in SDNN = ms  HRV in HF = ms^2^ and n.u.  HRV in LF = ms^2^ and n.u.  HRV in total power = ms^2^ | Eucapnia vs. isocapnic hypoxia vs. hypercapnic hyperoxia vs. hypercapnic hypoxia | Same as above |  |  |
| HRV indices were different between women and men during eucapnia, isocapnic hyperoxia, and hypocapnic hyperoxia. | HRV indices were not different between women and men during eucapnia, isocapnic hyperoxia, and hypocapnic hyperoxia. | HRV in women and men during eucapnia, isocapnic hyperoxia, and hypocapnic hyperoxia. | Same as above | Same as above | Same as above | **HRV in:**  **RMSSD in women vs. men:**  **P=0.990**  **SDNN in women vs. men:**  **P=0.685**  **HF (m^2^) in women vs. men:**  **P=0.950**  **LF (m^2^) in women vs. men:**  **P=0.496**  **Total Power in women vs. men:**  **P=0.701**  **HF (n.u.) in women vs. men:**  **P=0.438**  **LF (n.u.) in women vs. men:**  **P=0.397**  **LF/HF in women vs. men:**  **P=0.453** | Same as above | Same as above | Women vs Men | Same as above |  |  |

*You may use multiple lines for the same question to indicate multiple comparisons

** Authors may wish to make the text bold where p is considered significant against a stated confidence limit
